# PTEN: Multiple Functions in Human Malignant Tumors

**DOI:** 10.3389/fonc.2015.00024

**Published:** 2015-02-16

**Authors:** Michele Milella, Italia Falcone, Fabiana Conciatori, Ursula Cesta Incani, Anais Del Curatolo, Nicola Inzerilli, Carmen M. A. Nuzzo, Vanja Vaccaro, Sabrina Vari, Francesco Cognetti, Ludovica Ciuffreda

**Affiliations:** ^1^Division of Medical Oncology A, Regina Elena National Cancer Institute, Rome, Italy

**Keywords:** PTEN, cancer, subcellular localization, PHTS, PI3K

## Abstract

PTEN is the most important negative regulator of the PI3K signaling pathway. In addition to its canonical, PI3K inhibition-dependent functions, PTEN can also function as a tumor suppressor in a PI3K-independent manner. Indeed, the PTEN network regulates a broad spectrum of biological functions, modulating the flow of information from membrane-bound growth factor receptors to nuclear transcription factors, occurring in concert with other tumor suppressors and oncogenic signaling pathways. PTEN acts through its lipid and protein phosphatase activity and other non-enzymatic mechanisms. Studies conducted over the past 10 years have expanded our understanding of the biological role of PTEN, showing that in addition to its ability to regulate proliferation and cell survival, it also plays an intriguing role in regulating genomic stability, cell migration, stem cell self-renewal, and tumor microenvironment. Changes in PTEN protein levels, location, and enzymatic activity through various molecular mechanisms can generate a continuum of functional PTEN levels in inherited syndromes, sporadic cancers, and other diseases. PTEN activity can indeed, be modulated by mutations, epigenetic silencing, transcriptional repression, aberrant protein localization, and post-translational modifications. This review will discuss our current understanding of the biological role of PTEN, how PTEN expression and activity are regulated, and the consequences of PTEN dysregulation in human malignant tumors.

## What is PTEN?

PTEN stands for Phosphatase and TENsin homolog deleted on chromosome 10 and is a classical tumor suppressor gene located in the 10q23 region of chromosome 10 encoding for a 403-aminoacid multifunctional protein (predicted MW 47 kDa), which possesses lipid and protein phosphatase activities. PTEN was identified in 1977 by three independent groups: two groups used a positional-cloning approach, whereas the third group identified PTEN by a biochemical approach, which aimed to identify a gene encoding for a phosphatase with tensin and auxilin homology (1–3). The crystal structure of PTEN was resolved by Lee et al. in 1999, although two flexible regions of unknown function had to be deleted for technical reasons; crystal structure revealed the presence of a phosphatase domain, a C2 lipid membrane-binding domain, and a class I PDZ binding motif at the C-terminus, which recognizes target proteins (4). The PTEN gene is almost ubiquitously expressed in mammals throughout early embryogenesis (5); although the main protein subcellular localization is cytoplasmic and/or membrane bound, nuclear localization has been described and bears important functional consequences (6). In addition, it has been reported that the recently identified PTEN protein variants, such as PTEN-Long, can exit, exist, and function outside the cell in a *paracrine* type fashion (7).

**Figure 1:**
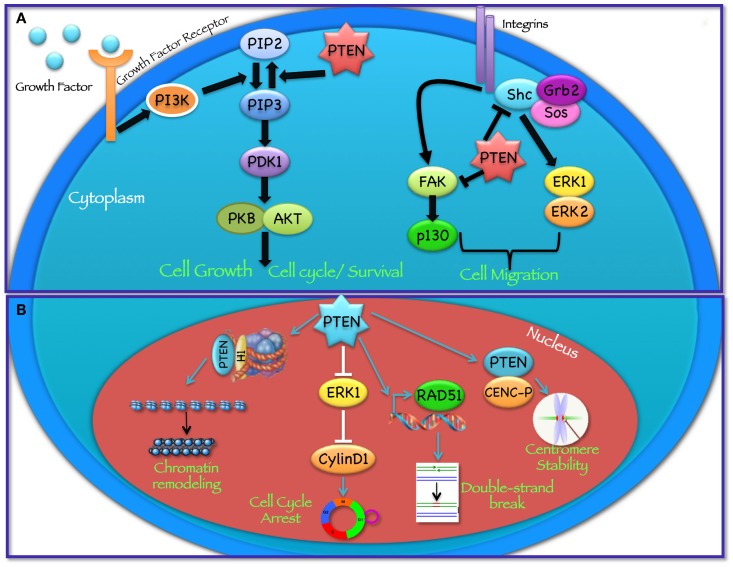
**Cytoplasmic and nuclear PTEN functions**. PTEN acts in regulating a wide spectrum of biological functions, at least in part determined by its subcellular localization. **(A)** In the cytoplasm PTEN dephosphorylates PIP3 to PIP2, thereby reversing the action of PI3K and hampering all downstream functions controlled by the AKT/mTOR axis, such as cycle progression, induction of cell death, transcription, translation, stimulation of angiogenesis, and stem cell self-renewal. In addition, through its protein phosphatase activity directed against FAK and SHC, PTEN modulates complex pathways affecting cell migration. **(B)** In the nucleus, PTEN cooperates in maintaining genomic integrity, repairing DNA double-strand breaks, controlling homologous recombination, and promoting ubiquitin-dependent degradation of oncoproteins such as PLK1 and AURK. In addition, PTEN controls cell-cycle progression by modulating ERK phosphorylation and cyclin D1 levels and regulates chromatin remodeling by binding to histone H1.

PTEN acts as a classical tumor suppressor, mainly involved in the homeostatic maintenance of the phosphatidylinositol 3 kinase (PI3K)/AKT cascade (Figure 1A). PI3K, a lipid kinase activated by receptor tyrosine kinases, G protein-coupled receptors, and RAS activation, converts the lipid second messenger phosphatidylinositol 4,5-bisphosphate (PIP2) to phosphatidylinositol 3,4,5-trisphosphate (PIP3); PIP3 recruits phosphatidylinositol-dependent kinase 1 (PDK1) and AKT to the plasma membrane, where AKT is phosphorylated on Thr308 by PDK1 and on Ser473 by the mammalian target of rapamycin (mTOR) complex 2 (mTORC2) (8, 9). By dephosphorylating PIP3 to PIP2, PTEN reverses the action of PI3K, thereby hampering all downstream functions controlled by the AKT/mTOR axis, such as cycle progression, induction of cell death, transcription, translation, stimulation of angiogenesis, and stem cell self-renewal (10–17). Even though the biological effects of PTEN are dominated by its ability to dephosphorylate lipid substrates, PTEN has also been reported to exhibit protein phosphatase activity, responsible for some of its biological effects, including inhibition of cell migration and cell-cycle arrest (18, 19). More recently, a growing list of functionally relevant, phosphatase-independent activities have been described (Table 1) (20, 21).

**Table 1:** **Selected cell functions controlled by specific PTEN activities/domains and their dependency on regulation of the PI3K pathway**.

Functions controlled by PTEN	PTEN activity involved	PTEN domain involved	PI3K/AKT dependency	References
Cell proliferation	Lipid phosphatase	Phosph/C2 (PBD?)	Yes	(4, 17)
Cell survival	Lipid phosphatase	Phosph/C2 (PBD?)	Yes	(4, 17)
Cell metabolism	Lipid phosphatase	Phosph/C2 (PBD?)	Yes	(4, 17)
Cell motility	Lipid and protein phosphatase	Phosph/C2	Partial^a^	(4, 17)
Angiogenesis	Lipid phosphatase	Phosph/C2 (PBD?)	Yes	(4, 17)
Chromosomal stability	Nuclear localization (phosphatase independent)	C-Tail		(4, 6, 17, 22)
Double-strand DNA breaks repair	Nuclear localization (phosphatase independent)	C-Tail	No	(4, 6, 17, 22, 23)
Cell-cycle progression	Lipid phosphatase	Phosph/C2 (PBD?)	Yes	(4, 17)
APC/C-CDH1-dependent PLK and AURK degradation	Nuclear localization (phosphatase independent)	Not determined	No	(6, 24)
Chromatin remodeling	Direct interaction with H1	C-Tail	No	(25)
JNK pathway activation	Not determined	Not determined	No	(26)
eIF2α- or MSP58-mediated transformation	Not determined (phosphatase independent)	C-Tail	No	(4, 17, 27, 28)
SRC activation	Protein phosphatase	Phosphatase	No	(17, 29)
Paracrine suppression of AKT activation	Secretion and lipid phosphatase	(PTEN-Long)^b^/Phosph/C2 (PBD?)	Yes	(7)
Mitochondrial metabolism and ATP production	Phosphatase	PTENα^c^/Phosph	No	(17, 30)

*^a^PTEN effects on cell motility are related to the PI3K-dependent formation/degradation of localized intracellular PIP3 gradients, as well as to the PI3K-independent dephosphorylation of FAK, SHC, and SRC family members*.

*^b^PTEN-Long is a translational variant of PTEN endowed with lipid phosphatase activity and the additional 173 aminoacids at the N-terminus constitute a secretion sequence that allows the protein to exit, exist, and function outside the cell*.

*^c^PTENα is a N-terminally extended form of PTEN that localizes to the mitochondria and regulates mitochondrial metabolism through the induction of cytochrome *c* oxidase activity and ATP production*.

PTEN function is commonly lost in a large proportion of human cancers through somatic mutations, gene silencing, or epigenetic mechanisms (Table 2). Tumor-associated mutations may occur in all PTEN domains, thus implying that each distinct protein region (and each related PTEN activity) may be pathologically relevant to cancer initiation and progression. In addition, emerging evidence shows that PTEN gene/protein dosage is quantitatively relevant during tumor development, as partial loss of PTEN function (haploinsufficiency) is sufficient to promote growth in some human malignancies. The mechanisms involved in regulation of PTEN dosage include methylation, micro-RNA (miR) or pseudogene expression, and protein phosphorylation (31–33). In this review, we will discuss the cellular functions controlled by PTEN, the molecular mechanisms of the subtle regulation of PTEN expression and function, and the role of its mutational/expression status in cancer.

**Table 2:** **Incidence and prognostic significance of PTEN alterations in PHTS and sporadic human cancers**.

Malignancy type	Increased risk in PHTS	Molecular mechanism(s) of PTEN alteration	Prognostic/therapeutic implications of PTEN loss	References
Breast cancer	Yes (85 vs. 12% LR)	Mutations <5%, LOH 40%, methylation 50%, and loss of expression ~40%	Resistance to endocrine and HER2-targeted therapy	(34–39)
Thyroid cancer	Yes (35 vs. 1% LR)	Homozygous deletion <10%, methylation >50%, rearrangement in most papillary thyroid carcinomas	PTEN loss cooperates with other genetic alterations and is more frequent in aggressive cancers (ATC)	(34, 35, 40)
Kidney cancer	Yes (34 vs. 1.6% LR)	Homozygous deletion or somatic mutations 1–5% of ccRCC and 6.4% of chRCC	High PTEN expression correlates with better DSS and better response to VEGFR-TKI	(34, 35, 41–43)
Endometrial cancer	Yes (28 vs. 2.6% LR)	Mutations 15–88% depending on specific subtype, methylation 18%, and loss of expression 20%	Favorable or unfavorable prognostic implications depending on mutation type and association with obesity and/or other factors	(34, 35, 44, 45)
Colorectal cancer	Yes (9 vs. 5% LR)	Up to 18% mutated and up to 19% LOH depending on tumor type, concomitant promoter hypermethylation	Inconsistent negative prognostic impact; lack of response to EGFR-targeted mAbs	(34, 35, 46–49)
Melanoma	Yes (6 vs. 2% LR)	LOH 30–60%, mutation 10–20% (metastases), and >50% frequent promoter methylation in patients with XP	Inconsistent association with prognosis; subcellular localization important; decreased response to BRAF-selective inhibitors	(34, 35, 50–53)
Glioma	Dysplastic gangliocytoma of the cerebellum in LD	LOH >70%, mutation 44% (coincident with LOH) and miR-26a amplification	Mutations associated with shorter OS	(34, 35, 54, 55)
Prostate cancer	NR	Homozygous deletion and mutation in up to 20%, miR-22 and miR-106b-25 cluster overexpression	Early recurrence after surgery, development of metastases, hormone refractoriness, and shorter survival	(34, 56–58)
Leukemia/lymphoma	NR	Deletion 10% of T-ALL and 27% mutation in T-ALL, aberrant RNA splicing in AML	Shorter survival and resistance to NOTCH inhibitors in T-ALL	(34, 59–70)
Lung cancer	Occasional	Mutations 6–9% (predominantly squamous), promoter methylation 24%, frequent miR-21 upregulation, and loss of PTEN 24–44%	Inconsistent association with poor prognosis, resistance to EGFR-targeted therapies	(34, 71–75)
Bladder cancer	NR	LOH 23%, homozygous deletion 6%, mutation 23% (late stage), and decreased or absent expression 53%	Significant association with recurrence in pTa and progression in pT1	(34, 76, 77)
Liver cancer	NR	Mutation ~5%, deletion or loss of expression ~50%, and protein expression downregulated by HBV and HCV viral proteins	Association with high tumor grade, advanced stage, high αFP expression; increased recurrence, shorter OS and possibly resistance to sorafenib	(34, 78, 79)
Pancreatic cancer	NR	Hetero or homozygous deletions 15%, loss of protein expression ~70% (exocrine); LOH ~50%, altered subcellular localization (endocrine)	Significantly increased recurrence and metastases, shorter OS (exocrine); negative prognostic impact modulated by PR and mTOR expression (endocrine)	(34, 80–82)
Phaeochromocytoma	NR	Mutations rare, LOH ~40%	More frequent in malignant versus benign lesions	(34, 83)

*PHTS, PTEN hamartoma tumor syndromes; LR, lifetime risk; LOH, loss of heterozygosity; ATC, anaplastic thyroid carcinoma; ccRCC, clear cell renal cell carcinoma; chRCC, chromophobe renal cell carcinoma; DSS, disease-specific survival; VEGFR-TKI, vascular endothelial growth factor receptor tyrosine kinase inhibitors; EGFR, epidermal growth factor receptor; mAbs, monoclonal antibodies; XP, xeroderma pigmentosum; LD, Lhermitte–Duclos syndrome; miR, misro-RNA; OS, overall survival; T-ALL, T-cell acute lymphoblastic leukemia; HBV, hepatitis B virus; HCV, hepatitis C virus; αFP, alpha fetoprotein; PR, progesterone receptor; mTOR, mammalian target of rapamycin*.

## Cellular Functions Controlled by PTEN

### PTEN-mediated regulation of metabolic pathways

The PTEN/PI3K pathway may influence key steps in metabolic pathways during cell proliferation and tumorigenesis. Recent data from two independently generated transgenic mouse models, based on two similar PTEN-containing bacterial artificial chromosomes (BAC), have shown that PTEN is involved in the control of metabolic pathways through PI3K-dependent and -independent functions (30, 84). Garcia-Cao et al. demonstrated that transgenic mice overexpressing PTEN show reduced body size, due to decreased cell number, increased energy expenditure, and reduced body fat accumulation. Cells derived from these mice show reduced glucose and glutamine uptake, increased mitochondrial oxidative phosphorylation, and resistance to oncogenic transformation. Ortega Molina et al. showed that mice carrying additional genomic copies of PTEN have increased energy expenditure and are protected from metabolic pathologies and cancer. Moreover, a recent human study demonstrates that PTEN haploinsufficiency is a monogenic cause of profound constitutive insulin sensitization that is apparently obesogenic. In particular, the authors demonstrated that patients who are heterozygous carriers of PTEN mutations, which cause Cowden syndrome, are at increased risk of obesity and cancer, but at decreased risk of diabetes due to enhanced insulin sensitivity (85). The role of PTEN in insulin-stimulated glucose uptake is controversial in the literature. Substantial evidence suggests a role for PTEN in the regulation of glucose uptake, due to its ability to modulate insulin signaling (86, 87). For example, Nakashima et al. have shown that PTEN overexpression in adipocytes inhibits insulin-stimulated, PI3K activation-dependent 2-deoxyglucose uptake, and glucose transporter type 4 (GLUT4) translocation, a key event in insulin signaling (88), which ultimately leads to decreased glucose cellular uptake. Conversely, Mosser et al. suggest that PTEN does not modulate GLUT4 translocation and metabolic functions of insulin under normal physiological conditions (89). However, Morani et al. using genetic manipulations of PTEN expression, have shown the involvement of PTEN in the regulation of membrane expression of glucose transporter type 1 (GLUT1), suggesting that PTEN regulates glucose uptake, at least in transformed cells, such as thyroid cancer cells (90). On the other hand, PTEN is involved in the modulation of gluconeogenesis through inhibition of forkhead box O (FOXO) 1, peroxisome proliferator activated receptor (PPAR)γ, and PPARγ-coactivator 1a (84).

### PTEN role in cell motility

Among its many functions, PTEN plays an important role in the regulation of cell motility, particularly, in controlling the directionality of chemotaxis. Studies conducted in Dictyostelium over the past years suggest that both PI3K and PTEN activities, and the resulting formation of localized intracellular PIP3 gradients, are required during chemotaxis (91–93). In these studies, the authors show that during chemotaxis PIP3 levels are enriched at the leading edge of migrating cells, while PTEN relocalizes at the opposite side of the cell; as a result, when PTEN is lost, Dictyostelium cells have defects in polarization and chemotaxis (94, 95). Several *in vitro* studies have shown that PTEN phosphatase activity is required to regulate cellular migration with some reports indicating the involvement of the C2 domain and another implicating PTEN’s protein phosphatase activity (96–99). In particular, it has been shown that PTEN overexpression is able to inhibit the spreading of glioblastoma cells and the migration and spreading of fibroblasts. On the other hand, knockdown of endogenous PTEN expression enhanced cell migration in fibroblasts possibly through the involvement of focal adhesion kinase (FAK) (100): PTEN dephosphorylates FAK, a cytoplasmic phosphoprotein activated by integrins, thereby inhibiting cell migration. PTEN may also dephosphorylate SHC, thus inhibiting downstream pathways, including the mitogen activated protein kinase (MAPK) pathway, which leads to the modulation of cell motility (Figure 1A). Recent studies, aimed at understanding the role of PTEN in cell motility, suggest that both lipid and protein phosphatase activities contribute to PTEN-mediated regulation of migration, through interactions with Rac1 and the SRC family kinase, FYN (101).

### PTEN and angiogenesis

Recent studies performed in glioblastoma cell lines suggest that PTEN is potentially important in the control of angiogenesis within brain tumors. In fact, reconstitution of PTEN expression in the PTEN−/− U87 MG glioma model caused a dramatic decrease in tumor growth *in vivo* and increase in mice survival, without significantly effecting the proliferation of these cells *in vitro*. The observed effect was due to the induction of thrombospondin-1, a negative regulator of angiogenesis, consequently reducing recruitment of blood vessels to the tumor (102). In addition, PTEN loss can also facilitate glioma growth by promoting HIF1 expression and activity (103). In both cases, PTEN’s ability to counteract neo-angiogenesis appears to be related to its lipid phosphatase activity and to suppression of signaling through the PI3K/AKT/mTOR axis. Our group and others have indeed demonstrated that the mTOR/eIF4E axis downstream of PI3K, plays a crucial role in the regulation of vascular endothelial growth factor (VEGF) production in breast cancer cells through increased hypoxia inducible factor 1α (HIF1α) expression and transcriptional activation. This suggests that the anti-tumor activity observed after PI3K (and especially mTOR) cascade inhibition may be due, at least in part, to the anti-angiogenic effects observed with mTORC1 inhibitors, such as temsirolimus (104). Along these lines, recent studies from our group have shown that the anti-angiogenic activity observed with MEK inhibitors in melanoma models *in vitro* and *in vivo* (105) is due, at least in part, to MEK inhibition-induced upregulation of PTEN expression. Indeed, in cell lines in which PTEN function is offset by the expression of a dominant negative PTEN mutant (DN-PTEN), the effects observed on VEGF production after MEK inhibitor treatment are partially blocked (106).

### Role of PTEN in the nucleus

PTEN plays an important tumor suppressor role in the nucleus (Figure 1B) and the absence of nuclear PTEN is associated with more aggressive cancers (107–109). Recent findings have shown that the PTEN protein is present in the nucleus of cell lines and tissue cells, even if the mechanisms of PTEN nuclear localization have yet to be fully elucidated (109–112). Given the absence of a classical nuclear localization signal sequence (NLS) or nuclear export sequences (NES), numerous molecular mechanisms have been proposed, including simple diffusion, active shuttling by the RAN GTPase, or phosphorylation-dependent shuttling and monoubiquitylation-dependent import (6, 113). Trotman et al. have demonstrated that PTEN nuclear import is mediated by its ubiquitination by the E3 ligase, neural precursor cell expressed, developmentally downregulated-4-1 (NEDD4-1) (114). Recently, sumoylation has also been reported to mediate PTEN nuclear retention (115). Within the nucleus, PTEN has been shown to interact with the centromere specific binding protein C (CENP-C) to modulate centrosome stability (22). Moreover, PTEN is involved in DNA-damage responses through upregulation of the transcription of Rad51, a key component of the homologous recombination system, which repairs DNA double-strand breaks (22, 23). In addition, nuclear PTEN controls cell-cycle progression by decreasing cyclin D1 levels in the nucleus and regulates cellular senescence through anaphase promoting complex (APC)-CDH1-mediated protein degradation (6, 24) (Figure 1B). Several reports suggest that nuclear PTEN functions may be independent of its catalytic activity and may be regulated by PTEN physical interaction with nuclear target proteins, such as p53, microspherule protein 1 (MSP58; also known as MCRS1) or HIF1α (27, 116, 117). Recent evidence also demonstrates that PTEN is involved in transcriptional regulation through the control of chromatin remodeling. Chen et al. have shown that PTEN physically associates with histone H1, through its C-terminal domain, to maintain a condensed chromatin structure. Loss of PTEN or its C-terminal portion promotes chromatin decondensation and gene activation (25).

### Alternative PTEN variants

Recent studies have led to the identification of new PTEN variants: PTEN-Long and PTENα. Hopkins et al. identified an alternative initiation codon, as compared to the canonical start site on the PTEN promoter, which predicts the expression of a longer PTEN variant. PTEN-Long contains a 173-amino acid domain at its N-terminus followed by the classical 403 amino acids of PTEN. PTEN-Long is a translational variant of PTEN endowed with lipid phosphatase activity and the additional amino acids constitute a secretion sequence that allows the protein to exit, exist, and function outside the cell (7, 118). Recent studies have reported that PTEN-Long can be detected in human serum and plasma and can act as a therapeutic factor involved in tumor regression (7, 119). Hopkins et al. have demonstrated that the purified PTEN-Long protein injected in mice is taken up by several tissues and antagonizes AKT signaling inducing an increase in blood glucose levels. In addition, they found PTEN-Long has tumor-suppressive function inducing tumor cell death *in vitro* and *in vivo* (7). The molecular mechanisms involved in PTEN-Long regulation have yet to be defined, but the long variant can be coexpressed with the classical PTEN isoform. PTEN-Long is expressed in normal tissues such as the breast and brain, whereas its expression levels are lower in human breast tumors and mouse models of glioblastoma (7, 20, 118).

Recently, Liang et al. have identified another N-terminally extended form of PTEN (PTENα) that localizes to the cytoplasm and the mitochondria and is involved in the regulation of mitochondrial metabolism through the induction of cytochrome *c* oxidase activity and ATP production. Moreover, PTENα promotes energy production and interacts with canonical PTEN to increase PTEN-induced putative kinase 1 (PINK1) protein levels (120).

## Fine Tuning of PTEN Activity

PTEN dysfunction plays a crucial role in the pathogenesis of hereditary and sporadic tumors (34, 121). During tumor development, mutations, and deletions of PTEN lead to the inactivation of its enzymatic activity, with consequently increased cell proliferation and reduced cell death. In addition to genomic inactivation, many other pathogenic mechanisms involved in the repression of PTEN gene expression or in the aberrant subcellular compartmentalization of the protein are associated with tumorigenesis. Several different mechanisms are known to fine tune PTEN expression and function, including transcriptional regulation, post-transcriptional regulation by non-coding RNAs, post-translational modifications, and protein–protein interactions.

### Transcriptional regulation

PTEN transcription is negatively and positively regulated by different transcription factors. p53 can upregulate PTEN, resulting in a complex interplay between these two tumor suppressors (122). EGR1 binds to the PTEN promoter and upregulates PTEN expression in response to insulin-like growth factor-2 (IGF-2) stimulation or radiation (123, 124). PPARγ can also upregulate PTEN gene expression (125).

On the contrary, SNAIL, c-Jun, and nuclear factor kappa-B (NF-kB) have been reported to negatively regulate PTEN transcription (106, 126, 127). Active NOTCH1 has been reported to act as both a negative and a positive regulator of PTEN transcription through interacting with MYC and CBF-1, respectively (128, 129). Our group and others have shown that c-Jun is involved in the regulation of PTEN expression (106, 130, 131). In particular, our group has demonstrated that PTEN is a target of a re-wired MEK–ERK–c-Jun survival pathway, occurring in BRAF-mutant melanoma, but also in other malignant tumors, as well as in normal fibroblasts. Indeed, upon genetic and/or pharmacologic modulation of ERK activity, c-Jun and PTEN are counter-regulated with strict time/dose dependency. Moreover, ERK-independent genetic modulation of c-Jun expression exerts the same effects on PTEN regulation and expression of a constitutively active MEK/ERK fusion protein in non-transformed fibroblasts concomitantly upregulating c-Jun and downregulating PTEN expression. These data reveal that ERK-dependent regulation of PTEN expression, occurring at least in part through c-Jun-mediated transcriptional repression, is a physiological regulatory mechanism, which takes place in both normal and cancer cells of different histological origin (106).

### Post-transcriptional regulation

micro-RNA contribute to the regulation of PTEN expression in many tumors. In fact, it has been demonstrated that the oncogenic miR-21, one of the most frequently upregulated miRs in cancer, directly targets and downregulates PTEN in specific cancers, including hepatocellular, ovarian, and lung cancer (132, 133). Recently, it has been shown that miR-25 controls PTEN levels in human tumors and contributes to experimental tumorigenesis (106, 134). As it has been discussed above for the involvement of c-Jun, miR-25 provides another interesting link between the MEK/ERK and PI3K/PTEN/AKT/mTOR pathways. Our group has recently shown that miR-25 expression levels are controlled by ERK activation status, which in turn regulates PTEN protein levels in melanoma cells. The MYC oncogene also may downregulate PTEN through increased expression of miR-19 (135).

In recent years, new findings have lead to hypothesize that both non-coding and protein-coding genes possess a novel mRNA-dependent non-coding function that enables them to act as a decoy to block the effect of specific miR on other RNA in a model termed the competing endogenous RNA (ceRNA) hypothesis (17, 134). This appears to be the case for the PTEN pseudogene 1 (PTENP1) that shares significant sequence identity with PTEN mRNA in regions that harbor miR target sites. PTENP1 was found to regulate PTEN expression through sequestration of PTEN-targeting miR, thereby increasing the half-life of PTEN mRNA and increasing PTEN protein levels (17, 134).

### Epigenetic silencing

Epigenetic silencing of PTEN expression through aberrant methylation of the gene promoter or of histone modifications has been observed in many types of cancers (17, 34). Several studies have shown that silencing of PTEN transcription is often due to the presence of hypermethylated CpG islands in the PTEN promoter. Hypermethylation has been observed in colorectal, endometrial, breast, gastric, prostate, melanoma, and lung cancer (19, 50, 71, 136, 137). Lu et al. have demonstrated that the zinc-finger transcription factor sal-like protein 4 (SALL4) represses PTEN transcription by recruiting an epigenetic repressor complex (Mi-2/NuRD) (138). Histone deacetylase inhibitors, on the other hand, are able to increase PTEN transcription in fibroblasts (139).

### Post-translational modifications

PTEN function is also regulated by post-translational modifications, including phosphorylation, acetylation, oxidation, and ubiquitination. PTEN has six phosphorylation sites, which have been implicated in the modulation of its tumor suppressor functions, stability, and subcellular compartmentalization (140). Caseine kinase 2 (CK2) is a protein kinase that phosphorylates PTEN on Thr366, Ser370, Ser380, Thr382, Thr383, and Ser385. CK2-mediated phosphorylation stabilizes PTEN, but creates a closer conformation that decreases interactions with binding partners and reduces its plasma membrane localization (111, 141). PTEN can be also phosphorylated by LKB1 on Ser385, resulting in its inactivation (142). Glycogen Synthase Kinase 3β (GSK3β) phosphorylates PTEN at Ser362 and Thr366, decreasing its phosphatase activity (143). PTEN phosphorylation can also be mediated by the kinases RAK, ROCK, JNK, JNKK, and SRC (141, 143–145).

Another mechanism involved in PTEN regulation is through the ubiquitin/proteasome pathway. NEDD4-1 is an E3 ubiquitin–protein ligase involved in promoting PTEN ubiquitin-mediated degradation (146). Recent evidence has identified WWP2 as an additional E3 ubiquitin ligase, which mediates PTEN ubiquitination-dependent degradation. Amodio et al. have shown that in some tumors, such as non-small-cell lung cancer (NSCLC), PTEN downregulation via ubiquitin-mediated degradation is an important mechanism leading to loss of PTEN activity (147). Previous studies have suggested that PTEN functions can be regulated by acetylation. The acetyltransferase P300/CBP-associated factor (PCAF) has been demonstrated to acetylate PTEN at Lys125 and Lys128 sites, which, in turn, negatively regulate PTEN catalytic activity (148). Catalytic PTEN activity is also controlled by reactive oxygen species (ROS). In particular, recent studies have shown that ROS regulate PTEN activity by oxidative-stress-induced formation of a disulfide bond between the active site Cys124 and Cys7 (149).

### Protein-protein interactions

Some cellular proteins, including phosphatases, can affect PTEN functions either directly or indirectly via protein–protein interactions. Such protein–protein interactions may influence PTEN activity by modifying its conformation, stability, subcellular compartmentalization, and lipid membrane binding. For example, membrane-associated guanylate kinase inverted 2 (MAGI2) binding to PTEN increases its activity (150). Conversely, DJ1 (also known as PARK7: Parkinson protein 7) binds PTEN under oxidative conditions, thereby inhibiting its activity. DJ1 expression is associated with increased AKT activity and poor prognosis in different tumor types (151, 152). Some proteins that interact with PTEN are involved in its translocation across the cytoplasm and subcellular localization (21). Microtubule-associated Ser/Thr kinase 2 (MAST2) is another PTEN regulator. Terrien et al. have recently demonstrated that when MAST2 and PTEN form a complex, the phosphorylation of PTEN by MAST2 drastically increases and destabilization of this interaction promotes neuronal cell survival, through the alteration of PTEN intracellular trafficking (153). Zmajkovicova et al., have demonstrated that interaction between PTEN, MEK1, and MAGI1 is necessary for PTEN membrane recruitment and PIP3 turnover and AKT signaling (154). MEK1 and MAGI1 are both essential for complex formation. In fact, MEK1 binding to MAGI1 promotes both complex formation and PTEN translocation onto the membrane.

## PTEN Loss in Human Cancer

Germline mutations of PTEN are the underlying genetic causes of related disorders clinically referred to as PTEN hamartoma syndromes (PHTS) including: Cowden syndrome, Bannayan–Zonana syndrome, Lhermitte–Duclos syndrome, Proteus syndrome, and Proteus-like syndrome. Mutations responsible for these syndromes result in a non-functional or absent protein, which causes uncontrolled cell growth, leading to tumor (either benign or malignant) growth (34, 155–157). Cowden syndrome is the best-described syndrome within PHTS, with approximately 80% of patients having germline PTEN mutations. Patients with Cowden syndrome have a high risk for benign and malignant tumors of the breast (lifetime risk – LR-85%), thyroid (LR 35%), kidney (renal cell carcinoma – RCC-, LR 33%), and endometrium (LR 28%), which correspond to sporadic tumor types that commonly exhibit somatic PTEN inactivation (155, 157, 158) (Table 2). In addition, increased risk for colorectal cancer (9%) and melanoma (6%), previously not believed to be part of PHTS, have also been described (35). In addition to the well-characterized role of PTEN mutations in PHTS syndromes, new evidence shows that PTEN mutation is one of the most validated causes of autism spectrum disorders, intellectual disability, and extreme macrocephaly (159, 160).

The PTEN tumor suppressor is frequently lost, either partially or fully, from many sporadic tumor types. Somatic inactivation of PTEN occurs in a wide range of neoplastic diseases, including melanoma, glioblastoma, colon, and endometrial cancers (161, 162) (Table 2). More than a decade of research has expanded our knowledge on PTEN’s role in cancer. Experiments performed in transgenic mice have demonstrated that loss of both copies of the PTEN gene results in embryonic lethality, whereas PTEN heterozygous mutants develop a diverse range of dysplasias in a wide spectrum of tissues with high incidence of prostate and colon cancer (163, 164). Interestingly, recent studies highlighted a crucial, dose-dependent role of PTEN in cancer, showing that subtle reductions in active PTEN levels dictate cancer susceptibility in a dose-dependent manner (31, 165). However, PTEN loss alone is sufficient to cause tumorigenesis in some tissues but not in others, making the role of PTEN more ambiguous (34). Accordingly, these data strongly support the hypothesis that PTEN is haploinsufficient tumor suppressor and, although PTEN deletion alone has minimal effects, it frequently contributes to tumorigenesis in the context of other genetic alterations (31, 166, 167).

### PTEN role in the regulation of stem cell biology

Recent data have begun to shed light on the critical role of PTEN in stem cell maintenance and cancer-initiating cell biology (11, 168). The effects of PTEN loss in stem cells may be tissue-dependent. PTEN deletion in neuronal stem cells increases proliferation and maintains their self-renewing capacity. Similarly, PTEN loss causes increased proliferation, de-differentiation, and progression toward prostatic intraepithelial neoplasia in prostate stem cells (30, 59, 168, 169). On the contrary, in both melanocytes and hematopoietic cells, deletion of PTEN leads to normal stem cell exhaustion (170, 171). PTEN loss has been shown to enhance the number of tumor initiating cells in a mouse model of leukemia and in solid tumors, such as breast cancer (172, 173). The role of PTEN expression and function in the maintenance of lung and colorectal cancer stem cells (CSC) has not been extensively studied. However, it is worthy to note that PTEN expression is usually very low in both lung and colon CSC. Moreover, in colon CSC, PTEN expression is also strikingly upregulated during differentiation and by stimuli that inhibit CSC growth and tumorigenic activity, such as such as BMP4 treatment or thymosin β4 targeting (11, 174, 175).

### PTEN in melanoma

Loss of PTEN plays an important role in the development of 30–60% of melanomas, however, the mechanisms by which loss of this gene leads to tumor formation remain uncertain. Recent evidence has shown that decreased PTEN transcript levels were associated with PTEN promoter methylation in melanoma (34, 50). Wang et al. showed that more than 50% of the melanomas from patients with xeroderma pigmentosum display PTEN mutations, typically related to ultraviolet radiation exposure, highlighting the link between DNA-damage and PTEN mutations in this disease (51). Dankort et al. have demonstrated the ability of PTEN silencing to cooperate with BRAFV600E mutations in the genesis of metastatic melanoma (52). Several research groups have recently demonstrated that MEK blockade may induce compensatory signaling through PI3K pathway in melanoma, inducing its own resistance factors and explaining the highly variable clinical responses to MEK inhibition in different cellular contexts (106, 131, 176–178). Without a doubt, PTEN status is critical in determining the functional outcome of pharmacologic MEK inhibition in melanoma and in cellular contexts in which PTEN is genetically unaltered, MEK blockade induces a cross-talk mechanism that leads to PTEN protein induction, playing an important, albeit not exclusive, role in the anti-tumor and anti-angiogenic activities of MEK inhibitors (106). Consistently, PTEN loss impairs the anti-tumor activity of MEK inhibitors in preclinical models (106, 179, 180) and correlates with decreased efficacy of BRAF-targeted treatments in metastatic melanoma patients (53).

### PTEN in pancreatic cancer

Mouse genetic studies, supported a potential role for PTEN as a haploinsufficient tumor suppressor. Indeed, homozygous deletion of PTEN in the pancreas leads to metaplasia that may progress toward frank carcinoma in approximately 20% of cases in transgenic mice. Decreased PTEN expression has been demonstrated in pancreatic tumor cell lines, although deletion or mutations that cause PTEN loss of activity have not been detected with significant frequency in human pancreatic ductal adenocarcinoma (PDAC) (80). In particular, Perren and collaborators have shown that, although PTEN is not mutated in pancreatic cancers, its subcellular localization may decrease its function (81). Ying et al. have recently documented a strong cooperative interaction between KRASG12D and PTEN loss in promoting metastatic PDAC (82).

### PTEN in colorectal cancer

PTEN mutations are relatively prevalent in colorectal cancer and constitute potential markers of response to EGFR and MAPK inhibitor-based therapies. In fact, PTEN loss or inactivating mutations are found in a variable proportion (5–30%) of sporadic colorectal cancers (46, 47, 181). Interestingly, studies performed in *in vivo* model systems show that PTEN reactivation in a colorectal cancer (CRC) cell line exhibiting PTEN loss reduces its metastatic capability without affecting primary tumor formation. Moreover, PTEN reactivation also changed the organotropic homing from liver and lung metastasis to liver-only metastasis (182). Importantly, Razis et al. have shown that PTEN levels are predictive of cetuximab efficacy in CRC models with activated EGFR signaling and wild type KRAS/BRAF status and in the presence of an intact PI3K/AKT pathway (48).

### PTEN in lung cancer

PTEN mutations occur at a low frequency in NSCLC and in small-cell lung cancer (SCLC), with the notable exception of squamous cell carcinoma of the lung, in which PTEN is mutated in 6–9% of the cases and significantly altered in up to 15% of cases, taking into account loss of expression as well (72). However, when detected, PTEN mutations appear to exert an effect on the therapeutic response to EGFR/PI3K pathway inhibitors (73). Since loss of PTEN protein expression is found in 24–44% of NSCLC (74), other mechanisms to decrease PTEN expression and function could be relevant in lung cancer. For example, epigenetic silencing may partially explain PTEN loss in cases when PTEN mutations or homozygous deletions are absent (71, 75). Soria et al. have suggested that 24% of early NSCLC samples lack PTEN expression, which correlated with PTEN promoter methylation (71). In addition, Zhang et al. have shown that levels of miR-21 were upregulated in lung cancer compared with normal lung tissue and correlate with a reduction in PTEN mRNA levels in advanced tumor stage (133).

### PTEN in breast cancer

Germline PTEN mutation in Cowden syndrome has a predisposition to breast cancer, where female CS patients have up to 85% LR of developing breast cancer (155, 157, 158). In sporadic breast carcinomas, the frequency of PTEN loss is 30–40% (36). Epigenetic aberrations also may cause a decrease in PTEN levels, strongly correlating with tumor stage and grade with complete loss occurring more frequently in metastatic than in primary tumors (76, 163, 167, 183). In addition, PTEN mutations may overlap with other mutations including human epidermal growth factor receptor 2 (HER2) and loss of a single PTEN allele has been shown to accelerate tumorigenesis in HER2-overexpressing breast tumors (184). Early studies by Nagata et al. show that, in addition to antagonizing HER2-driven tumorigenesis, PTEN also sensitizes breast cancer to trastuzumab treatment (37). In fact, PTEN loss and PTEN-independent activation of the PI3K pathway were identified as a major determinant of trastuzumab resistance in preclinical models and clinical samples as well (37–39, 185).

### PTEN in leukemia

Although mutations of the PTEN gene appear to be generally rare in hematological tumors (59), its functional inactivation is frequently observed in several hematopoietic neoplasms (60–62, 186). Experiments conducted in mice with inducible PTEN deletions have demonstrated that loss of PTEN drives proliferation of leukemia-initiating cells and promotes leukemogenesis. PI3K pathway blockade prevents leukemogenesis and restores the normal self-renewing capacity of hematopoietic stem cells (63, 64). Cheong et al. have shown that reduction of PTEN phosphorylation, associated with its inactivity, is observed in approximately 75% of acute myeloid leukemia (AML) patients (65). Another study has shown that aberrant PTEN transcripts are present in 24% of AML patients, 80% of cell lines, and 13% of normal controls analyzed (66). In chronic myeloid leukemia (CML), the BCR–ABL fusion protein mediates the exclusion of PTEN from the nucleus. Such effect is reversed upon inactivation of BCR–ABL by imatinib treatment, which restores PTEN physiological localization localization (67). Recently, it has been shown that PTEN loss accelerates T-cell acute lymphoblastic leukemia (T-ALL) onset, producing multiclonal tumors (68, 69). NOTCH1 receptor may inhibit PTEN expression through the HES-1 transcription factor and this may in turn lead to AKT activation and resistance to glucocorticoids (70). Indeed, T-ALL with PTEN loss are resistant to NOTCH1 inhibitors while they are sensitive to AKT inhibitors (68).

## Discussion and Conclusion

PTEN is an extremely powerful and multifaceted tumor suppressor functionally involved in many different “hallmarks” of cancer. The main mechanism by which PTEN activity restrains cancer development and progression remains its ability to downmodulate signaling through the PI3K pathway, thereby indirectly inhibiting AKT downstream targets, such as GSK3, FOXO, B cell lymphoma 2 (BCL-2) antagonist of cell death (BAD), the E3 ubiquitin–protein ligase MDM2 and p27, which control survival, cell proliferation, angiogenesis, and cellular metabolism (187). On the other hand, the mTORC1 arm of the PI3K/AKT/mTOR pathway is also activated in response to the loss of PTEN inhibitory activity, resulting in the phosphorylation of p70 ribosomal protein S6 kinase (S6K; also known as RPS6K) and inhibition of 4E-binding protein 1 (4EBP1; also known as eIF4EBP1) to activate protein translation (188, 189) leading to the enhanced translation of specific mRNAs that are crucial for cell growth and proliferation. Recently, 4EBP1 has indeed emerged as a key negative regulator of cell proliferation downstream of mTORC1, and its inactivation may directly promote the growth of sporadic cancers (190, 191).

As discussed above, novel tumor-suppressive functions of PTEN, independent of its lipid phosphatase activity and ability to keep the PI3K/AKT/mTOR pathway at bay, have recently emerged. Among these, functions related to PTEN nuclear localization appear to be particularly interesting (Figure 1B). Indeed, PTEN cooperates in maintaining genomic integrity, repairing DNA double-strand breaks, controlling homologous recombination, and promoting ubiquitin-dependent degradation of oncoproteins such as polo-like kinase 1 (PLK1) and Aurora kinases (AURK). This is particularly important, as loss of these PTEN activities may actually crucially sensitize tumor cells to the cytotoxic action of inhibitors of the DNA repair enzyme poly (ADP-ribose) polymerase (PARP) and have important implications in tumor cell sensitivity to PLK and AURK inhibitors (24, 192–194). Similarly, the phosphatase-independent role played by PTEN in controlling non-canonical signaling pathways, such as the JNK pathway (26), the eukaryotic translation initiation factor 2α kinase 2 (eIF2αK2; also known as PKR) – eIF2α phosphorylation cascade (28) or MSP58-mediated cellular transformation (27), and its ability to dephosphorylate protein substrates, such as FAK (195), cAMP responsive-element-binding protein (CREB) (196), and the non-receptor Tyr kinase SRC (29), may have important implications for the development of rational pharmacological combinations simultaneously targeting the PI3K/AKT and other relevant pathways.

Along these lines, another interesting indication toward the possible therapeutic exploitation of PTEN loss in human cancer is the observation that PTEN critically lies at the intersection of two major survival/proliferation pathways, the canonical PI3K/AKT/mTOR pathway (of which PTEN is an integral part), and the RAS/MEK/ERK pathway (11). Our group has recently shown that MEK inhibition restores PTEN expression in tumor cells with an intact PTEN gene by inhibiting a re-wired MEK–ERK–c-Jun/miR-25 survival pathway. Under these conditions, combined MEK/mTOR blockade may exert frank antagonistic effects in terms of inhibition of VEGF production and tumor cell growth (106). Conversely, loss of PTEN activity marks a functional state of relative resistance to the growth-inhibitory and anti-angiogenic activity of MEK inhibitors (53, 106, 179, 180), raising the interesting hypothesis that combined pharmacological inhibition of the MEK/ERK and PI3K/AKT/mTOR may result in highly synergistic anti-tumor activity selectively in PTEN-null tumors (11, 106, 197).

Synthetic lethality-based and rational combinatorial strategies highlighted above are two of the possible approaches to address the problem that usually happens with loss-of-function “oncogenic drivers,” restoring the oncosuppressive activity of a missing PTEN gene/protein by pharmacological means may be difficult. However, given the importance of non-genomic mechanisms regulating PTEN expression in those cases where the PTEN gene is conserved, epigenetic therapy through pharmacological modulation of histone acetylation status or promoter methylation, as well as inhibition of signaling pathways that are known to regulate PTEN expression, are particularly attractive, alone or in combination with inhibitors of other signaling pathways, whose activation is known to cooperate with PTEN loss in driving the different aspects of the neoplastic phenotype (198).

Finally, identification of clinical situations in which loss of PTEN activity is a major driving force, and hence PTEN-based therapeutics may be exquisitely effective, is cumbersome. The exceptionally complex regulation of PTEN activity calls for a more comprehensive assessment of PTEN status in human tumors. This should encompass sequencing the entire PTEN gene, transcriptional, and protein expression analysis, as well as assessment of post-translational modifications and subcellular localization. An alternative possibility could be to develop functional (genomic, transcriptional, or proteomic) “signatures” of loss of PTEN function. Although, such approach would require extensive validation to identify reliable surrogate(s) for PTEN loss, preliminary evidence suggests that “signatures” of PTEN loss may be developed and may identify patients/tissues with loss of PTEN function more efficiently than immunohistochemistry for the PTEN protein (26, 76).

Loss of the many PTEN activities remains a crucial event in the development and progression of a vast and ever increasing proportion of human cancers. Even though little progress has been made so far in developing agents to therapeutically enhance the tumor-suppressive functions of PTEN, there is little doubt that with the current rapid expansion of knowledge on PTEN functions and regulation, its exploitation for therapeutic purposes will soon become a reality for many cancer patients.

## Conflict of Interest Statement

The authors declare that the research was conducted in the absence of any commercial or financial relationships that could be construed as a potential conflict of interest.
